# Myocardial Feature Tracking Reduces Observer-Dependence in Low-Dose Dobutamine Stress Cardiovascular Magnetic Resonance

**DOI:** 10.1371/journal.pone.0122858

**Published:** 2015-04-07

**Authors:** Andreas Schuster, Matthias Paul, Nuno Bettencourt, Shazia T. Hussain, Geraint Morton, Shelby Kutty, Boris Bigalke, Amedeo Chiribiri, Divaka Perera, Eike Nagel, Philipp Beerbaum

**Affiliations:** 1 Department of Cardiology and Pulmonology, Georg-August-University and German Center for Cardiovascular Research (DZHK, Partner Site), Göttingen, Germany; 2 King's College London British Heart Foundation (BHF) Centre of Excellence; National Institute of Health Research (NIHR) Biomedical Research Centre at Guy's and St. Thomas' NHS Foundation Trust; Wellcome Trust and Engineering and Physical Sciences Research Council (EPSRC) Medical Engineering Centre; Division of Imaging Sciences and Biomedical Engineering; The Rayne Institute, St. Thomas´ Hospital, London, United Kingdom; 3 Cardiology Department—Centro Hospitalar de Gaia/Espinho, Porto, Portugal; 4 Joint Division of Pediatric Cardiology, University of Nebraska/Creighton University, Children’s Hospital and Medical Center, Omaha, Nebraska, United States of America; 5 Department of Cardiology, Campus Benjamin Franklin, Charité Berlin, Hindenburgdamm 30, Berlin, Germany; 6 King's College London BHF Centre of Excellence, NIHR Biomedical Research Centre and Department of Cardiology, Guy's and St. Thomas' NHS Foundation Trust, London, United Kingdom; 7 Department of Paediatric Cardiology, Hannover Medical School, Hannover, Germany; Semmelweis University, HUNGARY

## Abstract

**Objectives:**

To determine whether quantitative wall motion assessment by CMR myocardial feature tracking (CMR-FT) would reduce the impact of observer experience as compared to visual analysis in patients with ischemic cardiomyopathy (ICM).

**Methods:**

15 consecutive patients with ICM referred for assessment of hibernating myocardium were studied at 3 Tesla using SSFP cine images at rest and during low dose dobutamine stress (5 and 10 μg/kg/min of dobutamine). Conventional visual, qualitative analysis was performed independently and blinded by an experienced and an inexperienced reader, followed by post-processing of the same images by CMR-FT to quantify subendocardial and subepicardial circumferential (Ecc_endo_ and Ecc_epi_) and radial (Err) strain. Receiver operator characteristics (ROC) were assessed for each strain parameter and operator to detect the presence of inotropic reserve as visually defined by the experienced observer.

**Results:**

141 segments with wall motion abnormalities at rest were eligible for the analysis. Visual scoring of wall motion at rest and during dobutamine was significantly different between the experienced and the inexperienced observer (p<0.001). All strain values (Ecc_endo_, Ecc_epi_ and Err) derived during dobutamine stress (5 and 10 μg/kg/min) showed similar diagnostic accuracy for the detection of contractile reserve for both operators with no differences in ROC (p>0.05). Eccendo was the most accurate (AUC of 0.76, 10 μg/kg/min of dobutamine) parameter. Diagnostic accuracy was worse for resting strain with differences between operators for Ecc_endo_ and Ecc_epi_ (p<0.05) but not Err (p>0.05).

**Conclusion:**

Whilst visual analysis remains highly dependent on operator experience, quantitative CMR-FT analysis of myocardial wall mechanics during DS-CMR provides diagnostic accuracy for the detection of inotropic reserve regardless of operator experience and hence may improve diagnostic robustness of low-dose DS-CMR in clinical practice.

## Introduction

Cardiovascular magnetic resonance (CMR) plays an important role in the routine clinical management of patients with coronary artery disease (CAD). Its utility is increasing as it provides an assessment of myocardial function, hibernating myocardium and perfusion in a single examination [1–3].

CMR is considered the reference standard for wall motion analysis. It allows the visualization of left ventricular (LV) endocardial wall motion at rest, as well as during low and high dose dobutamine stress (DS) to assess myocardial hibernation and ischemia [3].

However the diagnostic accuracy of qualitative assessment (i.e. visual assessment) has been shown to be considerably operator dependent [4].

Myocardial tagging allows for quantitative assessment but requires the acquisition of additional tagging sequences and post processing [5, 6].

Recently CMR myocardial feature tracking (FT), a technique similar to echocardiographic speckle tracking, has been introduced [7]. CMR-FT allows the tracking of tissue voxel motion of cine-CMR images to derive wall mechanics and strain without acquisition of additional sequences [8]. CMR-FT has been validated against myocardial tagging with good agreement between CMR-FT and harmonic phase imaging (HARP) [9]. Recently, its use in determining quantitative wall motion assessment during intermediate dose DS in healthy volunteers and low-dose DS in patients with ischemic cardiomyopathy has been demonstrated [10, 11] with close agreement with myocardial scarring based on late gadolinium enhancement imaging (LGE) [11]. Those quantitative results should ideally be less-operator dependent and therefore useful in aiding the less experienced operator. We therefore designed a study to investigate how quantitative wall motion assessment with CMR-FT compares to conventional visual qualitative assessment, and whether the more inexperienced observer might benefit from a quantitative analysis when interpreting low-dose DS-CMR studies.

## Methods

We analyzed cine images of a cohort of consecutive patients (n = 15) referred for evaluation of ischemic cardiomyopathy. All patients enrolled in this study underwent CMR imaging on a 3 Tesla clinical MR scanner (Achieva, Philips Medical Systems, Best, The Netherlands). The St Thomas’ Hospital Research Ethics Committee approved the study protocol. All patients gave written informed consent before the CMR examination.

### Cardiovascular magnetic resonance

Imaging was carried out in the supine position using a 32-channel receiver coil. Cine images were acquired using a standard balanced steady state free precession (SSFP) sequence covering the whole LV (typical field of view 225 x 251 mm, matrix size 180 x 201). LV end-diastolic (EDV) and end-systolic volume (ESV), stroke volume (SV) and ejection fraction (EF) were measured at rest using Simpson’s method [12]. Volumes were adjusted to body surface area. Low-dose DS imaging was performed as previously described [13] with acquisition of 3 short axis slices covering 16 myocardial segments according to a recognized standard model [14]. Images were acquired at rest and during 5 and 10 μg/kg^/^min of dobutamine. All patient underwent late gadolinium enhancement (LGE) CMR in identical slice orientations between 10 and 20 min after administration of gadobutrol performed after the dobutamine study (0.2 mmol/kg Gadovist, Bayer, Germany) using standard methods (spatial resolution app. 1.7 × 1.7 × 8 mm^3^). Dysfunctional segments (mild hypokinesia or worse) were graded visually according to peak LGE transmurality in end-diastole as follows: (1) >0 to ≤25% (2) >25 to ≤50% (3) >50 to ≤75% and (4) >75% [15]. Scanning was performed according to standard protocols including a low dose DS test with an average duration of 40 minutes per scan [3]. A more detailed description of the MR imaging parameters has been reported previously [11].

### Visual Analysis

All images were analyzed by one experienced observer (7 years of MR experience) and a second observer with 2 years of imaging experience, including exposure to general MR imaging with basic skills in volumetry, perfusion and LGE imaging. The second observer had some experience in analysing resting wall motion however had very limited experience in analysing wall motion during low-dose dobutamine infusion to assess contractile reserve. For the purposes of this paper, the second observer is referred to as the inexperienced observer. These two independent observers analysed all cases according to a widely used scoring system: Wall motion was graded from SSFP images as normal (5), mildly hypokinetic (4), moderately hypokinetic (3), severely hypokinetic (2), akinetic (1) or dyskinetic (0) using the 16-segment model [14]. Images were analysed using CMR42 (Circle, Calgary, Canada, version 3.4). Both observers were blinded at each other’s results. Wall motion at rest was compared at the different stress levels in all segments. An improvement in wall motion by ≥1 category as determined by the experienced observer was regarded as indicative for inotropic reserve.

### Feature tracking

Myocardial wall mechanics were analysed using a dedicated software prototype (Diogenes MRI, Tomtec, Unterschleissheim, Germany).

**Fig 1:**
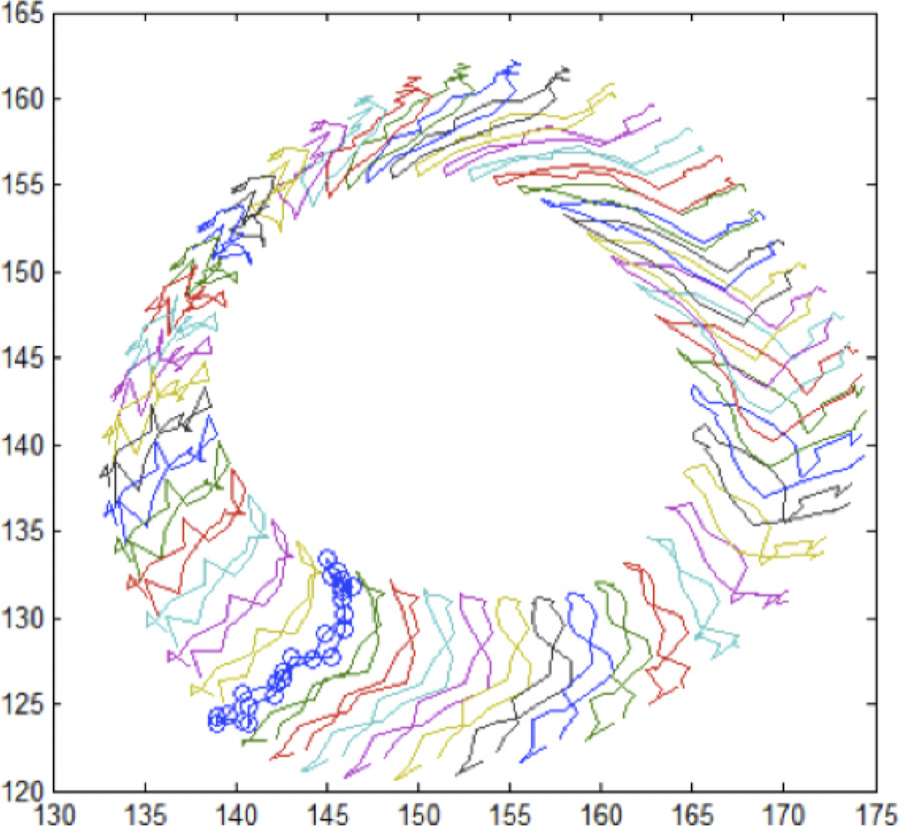
Example of the algorithm based feature tracking of a basal short axis slice. The figure shows the temporal evolution of the relative positions (pixel coordinates) of 48 control points per contour over the cardiac cycle, which in this example consists of 25 frames.

**Fig 2:**
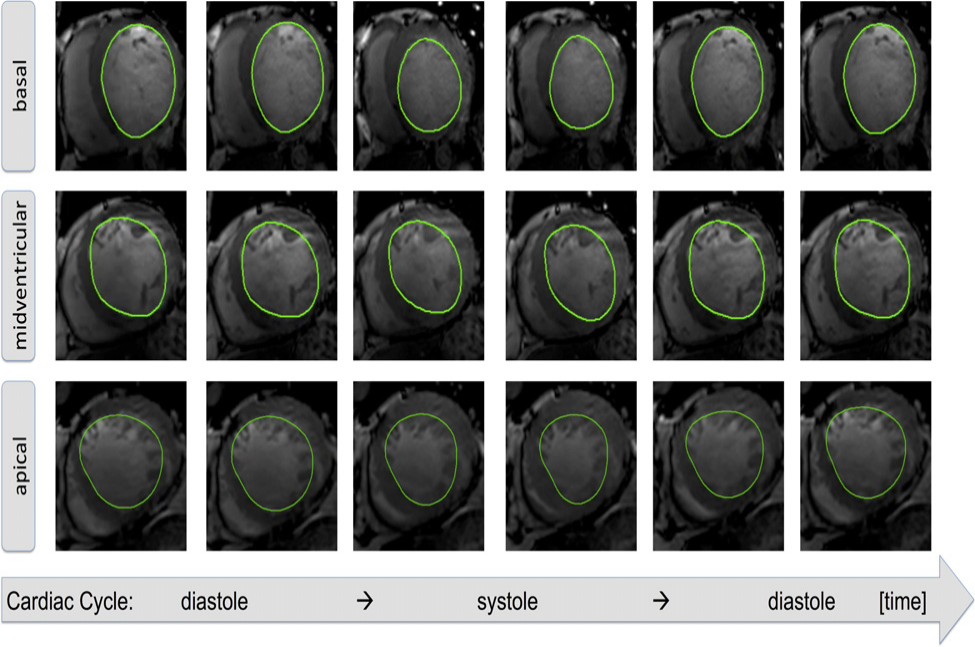
Contour Tracking in Short-Axis Orientation. The figure shows a representative example of the tracking in Short-Axis orientation of an ischemically damaged left ventricle (LV) at basal, midventricular and apical levels. The initial contour is set in end-diastole (left side of the rows) and after application of the feature tracking algorithm the contours (based on the underlying 48 control points) are automatically detected over the cardiac cycle.

CMR-FT was used to track tissue voxel motion of standard clinical short axis SSFP cine-MR images to derive circumferential and radial myocardial strain independent of additional sequences. This is done manually by delineating the endocardial and epicardial surface in an end-diastolic single phase. The software tracks a set of 2D points of the endocardial or epicardial surface. Strain is the percent change in length during a given time period and can be measured in the circumferential and radial direction. The algorithm uses 48 control points and their temporal evolution over a cardiac cycle is exemplarily illustrated in Fig 1. Short axis views at apical, midventricular and basal levels were used to calculate subendocardial (Ecc_endo_) and subepicardial circumferential (Ecc_epi_) and radial strains (Err) of all 16 LV segments. Endocardial contours were drawn manually in all analyzed slices by both observers (Fig 2). In case of insufficient border tracking the initial contour was corrected and the tracking algorithm was reapplied. All parameters were analyzed at rest, 5 and 10 μg · kg^-1^· min^-1^ of DS. Both observers manually allocated the RV superior septal insertion point of the LV to allow accurate segmentation and comparability between both analyses [14]. Strain values of segments with resting wall motion abnormalities, as visually defined by the experienced observer, were then compared between both observers at rest and with DS regarding the presence or absence of inotropic reserve.

### Statistics

Data analysis was performed with IBM SPSS statistics for Mac 19.0.0 (SPSS Inc., Chicago, Illinois, USA) unless otherwise stated. Continuous data are expressed as the mean ± SD. The paired samples t-test was used to compare measurements at rest and with DS after logarithmic transformation of the sample. The independent samples t-test was used to compare segments with and without inotropic reserve within one category. Observer specific wall motion scores were compared using the related samples Wilcoxon signed rank test. Receiver operator characteristics (ROC) analysis was used to determine the diagnostic accuracy and best threshold for all strain parameters and both operators to detect the presence of inotropic reserve as visually defined by the experienced observer.

To compare diagnostic accuracy we calculated the area under the curve (AUC) for each strain parameter and operator and compared these using the Delong test (Analyse-it for Microsoft Excel 2.20, Analyse-it Software, Ltd., Leeds, UK). A p-value of <0.05 was considered statistically significant.

## Results

The DS was well tolerated in all patients. While strain analysis could be performed in 208 of 240 of the segments (87%), the remaining 32 segments had to be excluded due to insufficient image quality. The reason for exclusion was severe breathing motion (n = 26) or inclusion of the outflow tract in the basal slice (n = 6). 141 segments with abnormal resting wall motion were included in the analysis with 66 segments showing LGE including 45 segments with scar transmurality below 50% (1–50%) [11]. Clinical characteristics of the study cohort are shown in Table 1.

**Table 1:** Subject Characteristics.

Patients	(n = 15)
Age (years)	69 ± 10
Male sex (%)	77
BMI (kg/m^2^)	27.2±5.5
1/2/3 vessel disease [%]	13 / 20 / 67
LV-ESV [ml/m^2^]	79±63
LV-EDV [ml/m^2^]	114±68
SV [ml/m^2^]	35±13
LV-EF [%]	35±10

The table shows demographics of the study population. Continuous variables are expressed as mean ± standard deviation. Volumes were adjusted to body surface area. BMI: Body mass index, ESV: endsystolic volume, EDV: enddiastolic volume, SV: stroke volume, LV: left ventricle

### Visual analysis

**Fig 3:**
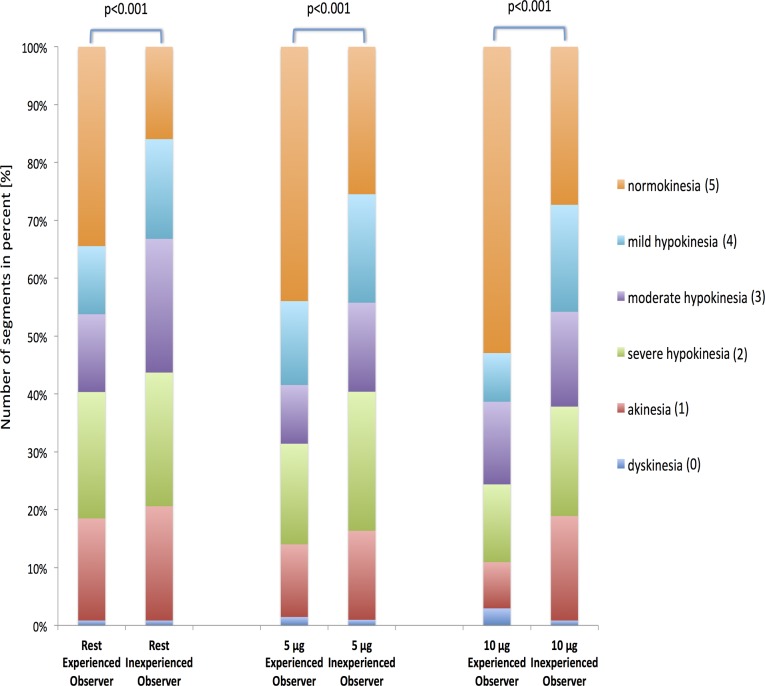
Comparison of visual analysis performed by one experienced and one inexperienced observer. The figure shows the results of the visual analysis for one experienced and one inexperienced observer. Wall motion was classified as normal (5), mild hypokinesia (4), moderate hypokinesia (3), severe hypokinesia (2), akinesia (1) or dyskinesia (0). Wall motion is presented regarding the individual stress level. Visual analysis was significantly different between the two observers.

Six segments had to be excluded from the visual analysis due to insufficient visualization with inclusion of the outflow tract within the basal slice. Visual analysis was performed in all other segments at all stress levels. Wall motion scoring was significantly different between the experienced and the inexperienced observer. This result was very obvious at rest but even more so at both stress levels (p<0.001 for each step, see Fig 3).

### Feature tracking

Results are displayed in Table 2. There was significant improvement of Ecc_endo_, Ecc_epi_ and Err with low dose DS as determined by the experienced observer. There was significant improvement in Ecc_endo_ however no improvement in Ecc_epi_ and Err as assessed by the inexperienced observer. Strain values in segments with inotropic reserve were different from segments without inotropic reserve at both stress levels for both observers (p<0.05).

**Table 2:** The table shows strain values of dysfunctional segments at rest, 5 and 10 μg/kg^/^min of dobutamine as determined by one experienced and one inexperienced observer.

Stress Level	Rest		5 μg/kg/min of dobutamine		10 μg/kg/min of dobutamine	
	Experienced	Inexperienced	Experienced	Inexperienced	Experienced	Inexperienced
**Ecc**_**endo**_	-9.1±6.5	-10.3±7.2	-11.7±7.5*	-12.5±8.4*	-12.8±8.9* ^#^	-11.6±8.1*
Visually +	-10.1±6.8	-12.0±7.3	-13.4±7.6*	-14.3±8.6*	-14.8±9.4* ^#^	-13.0±8.4
Visually -	-6.9±5.3	-6.5±5.6	-7.2±5.0	-7.9±5.5	-8.3±5.5*	-8.2±6.0
**Ecc**_**epi**_	-6.3±4.5	-7.0±5.5	-8.1±5.3*	-8.3±5.7	-8.8±5.9* ^#^	-7.7±5.3
Visually +	-6.4±4.6	-8.0±6.0	-8.9±5.4*	-9.3±5.8	-9.7±6.2* ^#^	-8.2±5.4
Visually -	-5.9±4.5	-5.0±3.6	-5.9±4.4	-5.8±4.6	-6.5±4.3	-6.4±4.7*
**Err**	10.8±8.8	11.8±9.8	13.9±11.1*	12.2±10.5	15.3±13.0*	12.6±10.2
Visually +	11.3±9.0	13.1±10.5	15.9±11.9*	14.3±11.2	17.6±14.2*	14.9±11.0
Visually -	9.8±8.6	8.8±7.6	8.5±6.5	6.7±5.8	10.3±8.0	7.4±5.7

Values for each parameter are presented for all dysfunctional segments and for subgroups with inotropic reserve (visually positive, Visually +) and without inotropic reserve (visually negative, Visually-). Values are expressed as mean ± standard deviation.

* indicating p<0.05 compared to rest

^#^ indicating p<0.05 in comparison between strain assessed at 5 and 10 μg/kg/min of dobutamine.

### Strain analysis to detect contractile reserve

**Fig 4:**
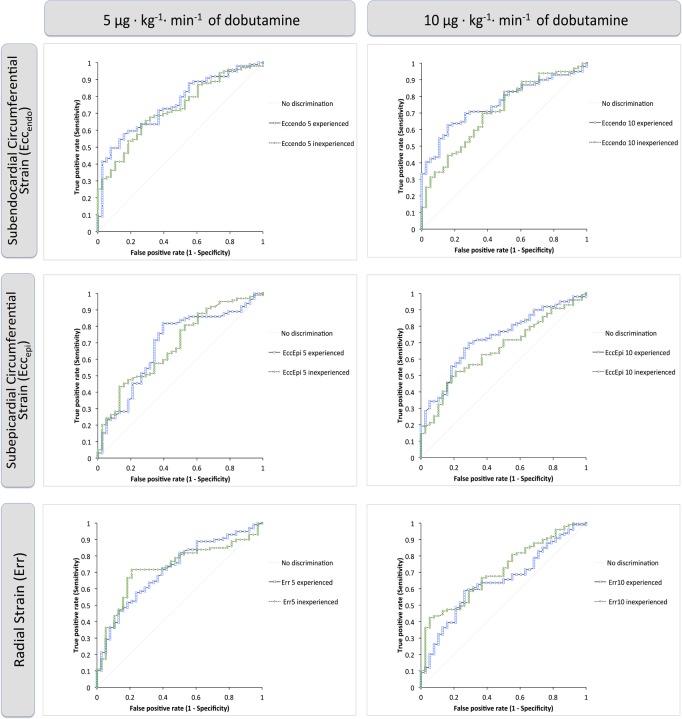
Receiver operator characteristics (ROC) for different strain values and observers. The figure shows the diagnostic accuracy of the different strain values derived from one experienced and one inexperienced observer to detect a visual improvement by ≥ 1 category as determined from the experienced observer’s visual analysis.

**Fig 5:**
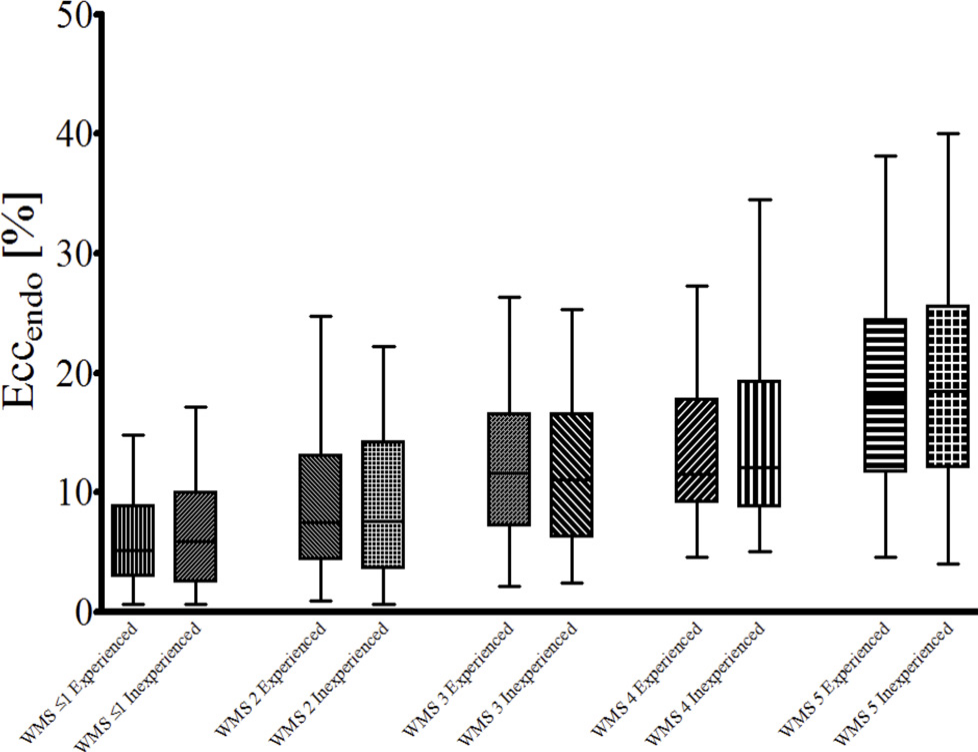
Relationship between strain values of the experienced and the inexperienced observer. Results show the strain values for (Ecc_endo_) of the experienced and the inexperienced observer according to the visual reference standard as defined by the experienced observers reading.

ROC curves were calculated for all strain parameters and each observer to assess the diagnostic accuracy of strain to detect inotropic reserve. Diagnostic accuracy was better for low-dose dobutamine as opposed to resting strain (Table 3). During DS, accuracy was best for Ecc_endo_ with an AUC of 0.76 and worst for Err with an AUC of 0.64 (during 10 μg/kg^/^min of dobutamine, respectively). Cut-off values that provided the optimal sensitivity and specificity to detect inotropic reserve are shown in Tables 3–5. Whilst ROC curves between both observers were different at rest, much in keeping with their disagreement seen at visual assessment, there was no difference between ROC curves of the experienced and the inexperienced observer during DS (Tables 3–5, Fig 4). There was no difference in strain values between observers when looking at subgroups according to wall motion abnormalities. Fig 5 shows this exemplarily for Ecc_endo_, representing the parameter with the best diagnostic accuracy in the ROC analysis. Furthermore, interestingly, the diagnostic accuracy between both observers did not differ in subgroups according to the transmurality of LGE (p>0.05). For segments with an scar transmurality below 50% (1–50%, n = 45) Ecc_epi_ reached slightly higher diagnostic accuracy for the experienced (AUC 0.84, 0.71–0.96) and the inexperienced observer (AUC 0.84, 0.71–0.97) as compared to Ecc_endo_ (Experienced: AUC 0.79, 0.61–0.97; inexperienced: AUC 0.82, 0.62–1) and Err (Experienced: AUC 0.69, 0.43–0.95; inexperienced: AUC 0.72, 0.47–0.98).

**Table 3:** The table shows the individual thresholds (cut-off) for left ventricular short-axis subendocardial circumferential strain (Ecc_endo_) as assessed by one experienced (Exp.) and one inexperienced (Inexp.) observer (Obs.) at rest and with 5 and 10 μg of dobutamine.

Strain	Obs.	Cut-Off	Sensitivity (CI)	Specificity (CI)	AUC (CI)	p-value
**Ecc**_**endo**_ **Rest**	Exp.	-7.54	0.68 (0.51–0.83)	0.54 (0.43–0.64)	0.62 (0.52–0.73)	<0.001
	Inexp.	-6.60	0.74 (0.57–0.87)	0.74 (0.64–0.82)	0.76 (0.67–0.85)		
**Ecc**_**endo**_ **5µg**	Exp.	-10.39	0.74 (0.57–0.87)	0.64 (0.53–0.73)	0.75 (0.67–0.84)	0.43
	Inexp.	-9.99	0.71 (0.54–0.85)	0.66 (0.55–0.75)	0.72 (0.64–0.81)		
**Ecc**_**endo**_ **10µg**	Exp.	-10.47	0.76 (0.6–0.89)	0.65 (0.54–0.74)	0.76 (0.68–0.84)	0.1
	Inexp	-9.81	0.71 (0.54–0.85)	0.56 (0.45–0.66)	0.71 (0.61–0.8)		

These cut-off values provided the optimal sensitivity and specificity, presented alongside their 95% confidence intervals (CI), to detect an increase in wall motion by at least 1 category as defined by the experienced observer’s visual analysis. The respective area under the curve (AUC) values are presented alongside their CI. P values refer to the difference between the AUC for the experienced and inexperienced observer.

**Table 4:** The table shows the individual thresholds (cut-off) for left ventricular short-axis subepicardial circumferential strain (Ecc_epi_) as assessed by one experienced (Exp.) and one inexperienced (Inexp.) observer (Obs.) at rest and with 5 and 10 μg of dobutamine.

Strain	Obs.	Cut-Off	Sensitivity (CI)	Specificity (CI)	AUC (CI)	p-value
**Ecc**_**epi**_ **Rest**	Exp.	-5.27	0.63 (0.46–0.78)	0.52 (0.41–0.62)	0.57 (0.46–0.68)	0.047
	Inexp	-4.60	0.61 (0.43–0.76)	0.61 (0.5–0.7)	0.64 (0.54–0.74)		
**Ecc**_**epi**_ **5µg**	Exp.	-7.35	0.71 (0.54–0.85)	0.55 (0.44–0.65)	0.69 (0.58–0.79)	0.96
	Inexp	-7.35	0.66 (049–0.8)	0.58 (0.47–0.68)	0.68 (0.58–0.78)		
**Ecc**_**epi**_ **10µg**	Exp.	-6.94	0.74 (0.57–0.87)	0.67 (0.57–0.76)	0.73 (0.64–0.82)	0.1
	Inexp	-7.15	0.79 (0.63–0.9)	0.53 (0.42–0.63)	0.66 (0.56–0.75)		

These cut-off values provided the optimal sensitivity and specificity, presented alongside their 95% confidence intervals (CI), to detect an increase in wall motion by at least 1 category as defined by the experienced observer’s visual analysis. The respective area under the curve (AUC) values are presented alongside their CI. P values refer to the difference between the AUC for the experienced and inexperienced observer.

**Table 5:** The table shows the individual thresholds (cut-off) for left ventricular short-axis radial strain (Err) as assessed by one experienced (Exp.) and one inexperienced (Inexp.) observer (Obs.) at rest and with 5 and 10 μg of dobutamine.

Strain	Obs.	Cut-Off	Sensitivity (CI)	Specificity (CI)	AUC (CI)	p-value
**Err Rest**	Exp.	9.46	0.58 (0.41–0.74)	0.5 (0.39–0.6)	0.52 (0.42–0.63)	0.1
	Inexp	7.85	0.61 (0.43–0.76)	0.6 (0.49–0.69)	0.61 (0.5–0.72)		
**Err 5µg**	Exp.	10.77	0.71 (0.54–0.85)	0.61 (0.5–0.7)	0.72 (0.63–0.81)	0.85
	Inexp	10.5	0.82 (0.66–0.92)	0.62 (0.51–0.71)	0.73 (0.64–0.82)		
**Err 10µg**	Exp.	10.79	0.69 (0.51–0.83)	0.61 (0.5–0.7)	0.64 (0.55–0.74)	0.2
	Inexp.	9.08	0.71 (0.54–0.85)	0.59 (0.48–0.68)	0.71 (0.61–0.80)		

These cut-off values provided the optimal sensitivity and specificity, presented alongside their 95% confidence intervals (CI), to detect an increase in wall motion by at least 1 category as defined by the experienced observer’s visual analysis. The respective area under the curve (AUC) values are presented alongside their CI. P values refer to the difference between the AUC for the experienced and inexperienced observer.

## Discussion

The current study demonstrates that CMR-FT can be used to interpret low-dose DS-CMR studies in a population of patients with ischemic cardiomyopathy, and that diagnostic accuracy of this novel technique for low-dose DS examinations appears to be independent of observer experience. This constitutes an important improvement of DS-CMR in comparison to the conventional visual assessment of wall motion abnormalities with its well-known great observer experience dependency, again demonstrated in the present study [4].

As the expertise required to sufficiently interpret low-dose DS-CMR studies is not present in all cardiac imaging centres, a quantitative less experience dependent approach may enhance the utility of the test. This is clinically relevant because the assessment of hibernating myocardium with low-dose DS-CMR has an important role particularly in segments where scar transmurality is below 50% and LGE imaging alone has limited diagnostic accuracy [13, 16]. The diagnostic accuracy of CMR-FT was similar between the observers in this clinically important subgroup in the current study. Recent publications show that a combination of different CMR parameters, most importantly LGE and inotropic stimulation with dobutamine, seem most efficient for this assessment [17, 18].

The aim of the current study was to investigate whether CMR-FT is useful to assist the less-experienced observer in interpreting low-dose DS-CMR cases. The presented data demonstrate good diagnostic accuracy for all three strain-parameters to detect inotropic reserve when applying individual cut-off values that have been previously developed and validated based on 3D myocardial tagging [19]. These accuracies were similar for an experienced and an inexperienced reader in the current study and therefore independent of observer experience. This holds true irrespective of whether segments with mild or more severe wall motion abnormalities or segments with different amounts of scarring or in the absence of scarring are studied. Conversely, our data confirms that visual analysis is highly operator dependent [4] and experience related. It maybe argued that the so-called inexperienced observer had some general CMR experience and therefore was not entirely inexperienced. We believe that this is a representation of a real world scenario with increasing numbers of physicians starting to use CMR but not necessarily use DS [20]. Many clinical centres use scar imaging as a first line approach to myocardial viability and hibernating myocardium [3] and only add a low-dose DS test when scar transmurality is below 50% [21]. Since low-dose stress testing is much less frequently performed than scar imaging particularly those observers who do not have much exposure to this test might benefit from additional quantification.

It is important to note that CMR-FT is not only an alternative to visual assessment of wall motion but provides a true quantitative measure of myocardial wall mechanics. This is underpinned by the current reference standard for myocardial deformation imaging, myocardial tissue tagging that has previously been demonstrated to improve diagnostic accuracy of DS-CMR in patients with suspected CAD as well as in patients with hibernating myocardium [5, 22–24]. However tagging is hampered by time constraints due to the need to acquire additional scans to perform tagging analyses, plus the associated extensive post-processing [5, 22]. CMR-FT wall mechanics compare well to tagging based harmonic phase imaging (HARP) wall mechanics in patients with Duchenne’s muscular dystrophy cardiomyopathy [9]. CMR-FT derived strain parameters can be used similarly to tagging at rest and with low-dose DS to assess hibernating myocardium in ischemic cardiomyopathy [11]. A good correlation between strain and transmurality of scarring as defined by LGE CMR imaging has been demonstrated [11]. Furthermore, inotropic stimulation has been shown to be quantifiable both in healthy volunteers [10] and patients with ischemic cardiomyopathy [11].

However, although the utility of these measurements has been demonstrated, the amount of variability in these measurements, present in both observers analyses, must be taken into consideration. This may make the detection of subtle changes more difficult. Indeed, the inotropic response was only detected within the Ecc_endo_ strain measurements by both observers, whereas Ecc_epi_ and Err did not show increased inotropy for the inexperienced observers strain analysis. There is evidence to suggest that strain in the circumferential direction is more reproducible than strain in the radial direction [25], which might explain the difference between Ecc_endo_ and Err however cannot explain the differences between the observers in Ecc_epi_. On the one hand, the tracking of the endocardium with the greater contrast between blood and heart tissue may be more robust as compared to the tracking of the epicardium. However, on the other hand the appearance of endocardial systolic trabecules may be a disadvantage of the endocardial tracking compared to the epicardial tracking. There is an intrinsic difficulty to estimate 2-dimensional strain metrics neglecting the out of plane movement that the tracked myocardial tissue experiences through the heart cycle. Even though this effect is less pronounced in ischemic cardiomyopathy where longitudinal shortening and through plane motion is reduced an attempt to reconstruct the true 3D deformations and strains would be highly desirable to overcome this fundamental limitation.

Notwithstanding these considerations, it is important to note that CMR-FT is equally feasible and reproducible both at 1.5 T and 3 T field strengths which is important for clinical interpretation of results acquired at different field strengths [26]. CMR-FT is a relatively novel method and its clinical value has not yet been fully established [27]. The considerable amount of variability that has been demonstrated before and can be appreciated by the relatively wide standard deviation of the individual measurements however suggests that CMR-FT may not yet be reliable enough for widespread clinical use at the current stage. Certainly the current algorithm needs to be further improved to reduce variability in order to allow inexperienced observers to clinically rely on this technique. There are certain angles of development that warrant further investigation such as defining the ideal spatial resolution to allow optimal border tracking. Furthermore improved temporal resolution may allow strain to be more accurately quantified. However, these refinements may be difficult to achieve in patients with ischemic cardiomyopathy where breath-holding is difficult particularly under dobutamine stimulation.

There is accumulating evidence that CMR-FT will be clinically useful for a variety of indications if these refinements can be made [9, 11]. Stanton et al. have demonstrated that echocardiography speckle-tracking derived global longitudinal strain is superior to EF and wall motion score index for the prediction of outcome [28] which has also been demonstrated in ischemic cardiomyopathy [29]. This finding has been recently reproduced in a cohort of patients with dilated cardiomyopathy using CMR-FT showing additive prognostic value of global longitudinal strain beyond established CMR parameters and biomarkers [30]. It is important to note, that echocardiography speckle tracking and CMR-FT based strain assessments have reasonable intermodality agreement particularly for global circumferential but also global longitudinal strain [31]. Therefore CMR-FT derived deformation data and echo speckle tracking derived data may be interchangeably assessed using both modalities to derive similar prognostic information. Novel CMR-FT applications including ventricular myocardial torsion and diastolic recoil as well as atrial performance analysis are being introduced that may provide additional tools for systolic and diastolic function quantification [32, 33]. Future work should aim to define specific indications for routine use of the available CMR-FT quantitative markers of myocardial deformation.

### Limitations

We have performed a proof of concept small feasibility investigation in 15 consecutive patients with ischemic cardiomyopathy. As a reference standard for inotropic reserve we used a visually determined increase of wall motion with dobutamine. Our findings need to be reproduced in prospective investigations that include a larger cohort and follow-up post revascularization data to provide a true gold standard.

### Conclusion

CMR-FT derived quantitative parameters appear to be less observer dependent than visual analysis during low dose DS-CMR in patients with ischemic cardiomyopathy. This is particularly relevant for less experienced observers when assessing hibernating myocardium. However the current algorithm needs to be further refined reducing variability in order to allow for CMR-FT to be fully established as a clinical tool. Should these refinements be implemented and findings of the current study be confirmed in prospective investigations, CMR-FT may emerge as an easy quantitative application providing true myocardial deformation information and thereby facilitating study interpretation of low dose DS-CMR and clinical decision-making.
